# Abbott’s History of the Rebellion

**Published:** 1863-06

**Authors:** 


ABBOTT'S HISTORY OF THE REBELLION.—We have received the first volume of this book; the second is to be published at the close of the war. We find it truthful, impartial, reliable, every way a true and faithful history of events as they have occurred. The illustrations are very fine, and the portraits of distinguished individuals are alone worth the price of the book. It contains about 500 pages, is tastily bound, and is a very desirable work for every one to possess. We are pleased with its spirit and style, and invite our readers to consider it favorably.

